# Rapid micro-assays for amylolytic activities determination: customization and validation of the tests

**DOI:** 10.1007/s00253-018-09610-0

**Published:** 2019-01-23

**Authors:** Monika Borkowska, Wojciech Białas, Monika Kubiak, Ewelina Celińska

**Affiliations:** Department of Biotechnology and Food Microbiology, Poznan University of Life Sciences, ul. Wojska Polskiego 48, 60-627 Poznań, Poland

**Keywords:** Alpha-amylase, Glucoamylase, Enzymatic assay, Thermalcycler, Micro-volume, Microassay

## Abstract

High-throughput function-based screening techniques remain the major bottleneck in the novel biocatalysts development pipeline. In the present study, we customized protocols for amylolytic activity determination (Somogyi-Nelson and starch-iodine tests) to micro-volume thermalcycler-based assays (linearity range 60–600 μM of reducing sugar, R^2^ = 0.9855; 0–2 mg/mL of starch, R^2^ = 0.9921, respectively). Exploitation of a thermalcycler enabled rapid and accurate temperature control, further reduction of reagents and samples volumes, and limited evaporation of the reaction mixtures, meeting several crucial requirements of an adequate enzymatic assay. In the optimized micro-volume Somogyi-Nelson protocol, we were able to reduce the time required for high-temperature heating sixfold (down to 5 min) and further increase sensitivity of the assay (tenfold), when compared to the previous MTP-based protocol. The optimized microassays have complementary scope of specificities: micro-starch-iodine test for endoglucanases, micro-Somogyi-Nelson test for exoglucanases. Due to rapid, micro-volume and high-throughput character, the methods can complement toolbox assisting development of novel biocatalysts and analysis of saccharides-containing samples.

**Electronic supplementary material:**

The online version of this article (10.1007/s00253-018-09610-0) contains supplementary material, which is available to authorized users.

## Introduction

Significant effort is now being pursued towards development of highly efficient, industrially relevant biocatalysts—either whole microbial cells or individual enzymes. The advent of novel high-throughput strategies supported by robust modular cloning techniques (Celinska et al. 2017; Celińska et al. 2018; Engler et al. 2008), efficient directed evolution approaches (Dragosits and Mattanovich 2013), and powerful genome editing tools (Sander and Joung 2014; Mans et al. 2015; Stovicek et al. 2015) allow for generation of numerous variants of the biocatalysts in relatively short time. In spite of great advancement within the field of the biocatalysts generation, high-throughput function-based screening techniques remain the major bottleneck in the novel biocatalysts development pipeline (Quin and Schmidt-Dannert 2012; Madhavan et al. 2017). The urgent need for powerful, high-throughput screening methodology was initially realized in the context of directed evolution strategies (Portnoy et al. 2011; Tizei et al. 2016), which generate large amounts of mutants with uncharacterized genetic background to be screened. Nonetheless, good laboratory practice concomitant with awareness of clone-to-clone variability requires screening of a sufficient number of clones—also those bearing rationally designed modifications, which in turn calls for efficient screening protocols, as well.

Recently, several interesting high-throughput screening approaches have been presented, like droplet-based microfluidic system for rapid encapsulation, cultivation, assaying and sorting of yeast cells expressing heterologous hydrolytic enzymes (Beneyton et al. 2017), protocols for high-throughput cultivation screening with real-time data acquisition (Back et al. 2016), fluorescence-activated cell sorting (FACS) of cell-surface displayed protein mutants (Olsen et al. 2000; Mattanovich and Borth 2006), or miniaturized potentiometric sensors (Sakač et al. 2011) or immunosensors (Della Ventura et al. 2017) for the determination of enzymatic activity. These techniques exhibit superiority in terms of high-throughput character, time-efficiency, accuracy, and precision; however, they require specialized equipment or specific structure of the DNA constructs, which might be considered as a kind of limitation. On the other hand, miniaturized enzymatic assays, conducted in micro-volume scale followed by automated measurement in a microplate reader, are repeatedly found a useful strategy in screening extensive libraries of the biocatalysts (Packer and Liu 2015). Amongst the most important advantages of the MTP (microtiter plate)-based screening, one can name higher accessibility to the required equipment, reagents and consumables, broad dynamic range allowing for detection of comparatively low improvements in the targeted enzyme function compared to other methods like colony screening assays (Kelly et al. 2008), similarity to a miniature cuvette systems, allowing for customization of standard enzymatic assays to the MTP format. Popularity of this approach is reflected by a number of regularly published new protocols describing customization of enzymatic assays to the MTP format, including industrially relevant enzymes such as lipases (Mustafa et al. 2016) or glycosidases—by adopting glucose assay (Visvanathan et al. 2016), Somogyi-Nelson method (Shao and Lin 2018), or 3,5-dinitrosalicylic acid (DNS) method (Goncalves et al. 2010), and cellulolytic enzymes (Xiao et al. 2005; King et al. 2009).

Glycosidases (glycoside hydrolases; including amylases, invertases, xylanases) are expected to progressively gain importance in the global industry due to their roles as catalysts in biorefining applications in the future bioeconomy. Currently, this group of enzymes is widely applied in various branches of the global market including food production and processing, degradation of plant biomass, or in the paper and pulp industry. Our recent studies were focused on expression of an insect (S*itophilusoryzae*) gene encoding alpha-amylase (*SoAMY*) and a fungal (*Thermomyceslanuginosus*) glucoamylase (*TlGAMY*) in an efficient expression host—a nonconventional yeast species *Yarrowia lipolytica* (Celińska et al. 2015, 2016b, 2017; Celińska et al. 2018). We have demonstrated that the recombinant alpha-amylase exhibits raw starch-digesting properties (Celińska et al. 2016a) which is one of the most desired, industrially relevant characteristics of the alpha-amylases (Sun et al. 2010). Alpha-amylases are endoglucanases that cleave internal α-1,4-glycosidic bonds in starch to produce shorter saccharides. In practice, another enzymatic activity is routinely used to achieve practically complete hydrolysis of the biopolymer—glucoamylase. Glucoamylases are exoglucanases releasing glucose monomers from the non-reducing end of starch. TlGAMY glucoamylase was proved to operate efficiently as a component of a consolidated biocatalyst system, constructed in *S. cerevisiae* (Favaro et al. 2015).

Assaying the hydrolytic activity of alpha- or glucoamylase may rely on either measuring an increase in the amount of released product (reducing sugars) or a decrease in the amount of the substrate. Protocols falling into the former category include commonly known assays employing 3,5-dinitrosalicylic acid (DNS) (Miller 1959) or alkaline copper and arsenomolybdate in a Somogyi-Nelson method (Nelson 1944; Somogyi 1952), whereas the latter category is represented by iodine-based test, where helical, undigested starch forms blue complexes with triiodide anions (Fuwa 1954). Starch-iodine test can be also easily adopted as a rapid and simple approach for preliminary screening of large libraries of microbial strains by cultivation of the microorganisms in starch-containing medium followed by staining with iodine. However, in such a format, the method renders only qualitative data, which is frequently not sufficient for accurate selection of the mutants/clones to be further studied. To address limited screening capacity of standard protocols and maintaining quantitative character of the obtained data, both the starch-iodine test (Xiao et al. 2006) and the Somogyi-Nelson reducing-sugar assay (Green et al. 1989; Shao and Lin 2018) were adopted to an MTP format.

In the present study, we aimed at customization of standard protocols of assessing amylolytic activity to microscale employing a thermalcycler apparatus, in order to further improve screening capacity of the protocols. Such an approach has been already successfully implemented to the DNS assay (Lucena et al. 2013). Exploitation of a thermalcycler in development of micro-volume enzymatic assays ensures rapid and sharp temperature control in the reaction tubes during incubation and boiling steps and further reduces the volumes of samples and reagents required for the reaction, when compared to the MTP format, as well as minimizes evaporation or leakage of the reaction mixture. Importantly, application of a thermalcycler allows for incorporation of a boiling step in the protocol (frequently crucial for the color development) which is not possible in the standard MTP-based protocols due to limited resistance of polystyrene MTP plates to elevated temperatures. We adopted the Somogyi-Nelson test (SNT) and starch-iodine test (SIT), which were in routine use in our laboratory in a macroscale, to the microassays conducted in a thermalcycler apparatus. While the SIT protocol could be easily customized to the micro-volume format by conducting several experiments concerning determination of linearity range of the method conducted at decreased volumes or investigation of potential changes in the absorbance spectrum, the SNT protocol required more extensive optimization studies. Established micro-volume protocols were subsequently thoroughly compared with respect to their performance towards purified enzymatic preparations and raw supernatant samples withdrawn from the cultures of recombinant yeast expressing *SoAMY* and *TlGAMY*. Moreover, the optimized microassays were compared with their macro-volume counterparts, to give better assessment of novelty and achievements of the current research.

## Materials and methods

### Reagents

Sodium molybdate dihydrate (Na_2_MoO_4_x2H_2_O), sodium sulfate (Na_2_SO_4_), sodium carbonate (Na_2_CO_3_), sodium bicarbonate (NaHCO_3_), sodium-potassium tartrate tetrahydrate (KOCOCH(OH)CH(OH)COONax4H_2_O), copper sulfate pentahydrate (CuSO_4_x5H_2_O), potassium iodide (KI), iodine (I_2_), hydrochloric acid (HCl), and glucose were all purchased from POCh (Gliwice, Poland). Sodium arsenate dibasic heptahydrate (Na_2_HAsO_4_x7H_2_O) and rice starch were purchased from Sigma (St. Louis, USA). The purified enzymatic preparation of the recombinant insect alpha-amylase (SoAMY), as well as the recombinant fungal glucoamylase (TlGAMY), was obtained and purified as described previously (Celińska et al. 2015, 2018).

### Strains, media composition, and culture conditions

*Yarrowia lipolytica* Po1g *(MatA, leu2-270, ura3-302::URA3, xpr2-332, axp-2; Yeastern Biotech Co., Ltd., Taiwan)* and Po1d *(MatA, leu2-270, ura3-302, xpr2-322)* and their respective descendants were used in this study. The strains were transformed with either pYLSC-*SoAMY*, pYLSC-*TlGAMY*, or Golden Gate Assembly cassettes bearing one of the heterologous genes, as described in (Celińska et al. 2016b, 2018). The heterologous genes encoding alpha-amylase from *S. oryzae* or glucoamylase from *T. lanuginosus* were codon optimized and cloned via corresponding method as described elsewhere (Celińska et al. 2016b, 2018). The *Y. lipolytica* recombinant strains were routinely maintained on YPD agar plates (g/L): yeast extract (BIOCORP, Warsaw, Poland), 10; bactopeptone (BIOCORP, Warsaw, Poland), 20; glucose, 20 (POCh, Gliwice, Poland), agar, 20 (BIOCORP, Warsaw, Poland). Production cultures were conducted in U-bottom microtiter plates with the well volume of 300 μl and the culture medium volume of 200 μl, in YPG medium (g/L): yeast extract, 10; bactopeptone, 20; glycerol (POCh, Gliwice, Poland), 20; at 30 °C, 250 rpm a rotary shaker (BIOSAN, Riga, Latvia) over 24 h. Prior to the enzymatic assays, the biomass was separated from the post culturing liquid by centrifugation (4 krpm, + 4 °C). For the macroassays, the supernatants of replicate cultures were pooled to obtain sufficient amount of crude enzymatic preparations. All the cultures were conducted in biological duplicate and at least technical duplicate.

### General procedure

In the current study, two standard tests—starch-iodine (SIT) and Somogyi-Nelson (SNT)—were customized to the thermalcycler-based formats. Schematic presentation of all the protocols in a form of diagrams can be found in Fig. S1. In the present study, two types of the enzyme-containing samples were used in the assays: (i) concentrated enzymatic preparation purified via affinity chromatography, (ii) raw culture medium supernatants containing secreted enzymes. Incubation time of the substrate and the enzyme-containing sample at 40 °C in the thermalcycler (microassays) or the water bath (macroassays) varied depending of the sample type: (i) 15–30 min for the purified enzymatic preparation, (ii) 60–120 min for the culture media supernatants. Such adjustment was necessary in order to fit the absorbance readouts into the linear range of the method. Noteworthy, this incubation time adjustments did not influence the final results expressed in activity units, as the activity units were normalized versus a time unit (1 min for all the assays).

#### Microassays

A Verity 96-well Thermal Cycler (Applied Biosystems, Foster City, USA) was used for incubation and heating of the samples. The enzymatic microassays were performed in 96-well semi-skirted PCR plates (4-titude, Wotton, UK) tightly covered with AxyMat silicone sealing mats (Axygen, Union City, USA). Twenty microliters of rice starch (2 mg/mL) in acetate buffer (100 mM, pH 5.0) was used as the substrate, and combined with an equal volume of the sample containing the enzyme (either purified preparation or crude culture medium supernatant). Substrate hydrolysis was conducted as described in sections for dedicated for microSNT and microSIT assays, respectively. Completed reaction mixtures (processed according to the protocols specific for a respective assay—SNT or SIT) were subsequently transferred into a transparent flat-bottomed 96-well assay microplate (Corning, NY, USA) and analyzed using a Tecan Infinite M200 automatic plate reader (Tecan Group Ltd., Männedorf, Switzerland), measuring the absorbance of the samples (wavelength 600 nm for SNT, and 580 nm for SIT). All the reactions using purified enzymatic preparation or crude medium were done in at least biological duplicate and each of them—in technical duplicate. Reactions performed with standard solutions were conducted in at least triplicate.

#### Macroassays

A laboratory water bath (BIOSAN, Riga, Latvia) and a thermalblock (BIOSAN, Riga, Latvia) were used for incubation and boiling of the processed samples. The assays were conducted in 2 mL Eppendorf tubes (Starstedt, Nümbrecht, Germany). In the macro-format, 500 μL of starch solution (2 mg/mL) in acetate buffer (100 mM, pH 5.0) was used as the substrate, and combined with 500 μL of the sample containing the enzyme (either purified preparation or culture medium supernatant). Substrate hydrolysis was conducted as described in sections dedicated for macroSNT and macroSIT assays, respectively. Absorbance of the completed reaction mixtures (processed according to the protocols specific for a respective assay—SNT or SIT) was measured using Analytik Jena Spectrophotometer (Analytik Jena, Jena, Germany) and WinASPEKT Software and compatible, standard 1.5 mL PS cuvettes (Starstedt, Nümbrecht, Germany).

### Somogyi-Nelson test

The concentration of reducing sugars was determined according to a modified Somogyi-Nelson method (Nelson 1944) versus a standard curve prepared with glucose (0.06–0.6 mM) under corresponding conditions. The test-specific reagents were prepared as follows: Nelson’s copper reagent I (g/ 800 mL): sodium carbonate anhydrous, 24, sodium bicarbonate, 16, sodium-potassium tartrate tetrahydrate, 12, sodium sulfate, 144; Nelson’s copper reagent II (g/200 mL): copper sulfate pentahydrate, 4, sodium sulfate, 36; prior the enzymatic reaction, the reagents I and II were mixed in proportion 4:1; arsenate-molybdate reagent (g/L): sodium molybdate dihydrate, 50, concentrated sulfuric acid, 42 mL, sodium arsenate dibasic heptahydrate, 6. The latter solution (Nelson’s copper reagent II) was incubated at 37 °C, over 24–48 h prior to use.

The initial optimization tests of the SNT protocol (boiling time, heating temperature, volume of the reagents, and standard curves for micro- and macro assay) were conducted using standard glucose solutions (0.06–0.6 mM; 0.0108–0.108 mg/mL). Absorbance spectrum for microSNT was analyzed using 0.6 mM glucose solution in a reaction mixture described hereafter (microSNT), making absorbance measurements in the range of λ = 500–750 nm in 10 nm intervals.

#### One activity unit

It was expressed as the amount of an enzyme that releases 1 mmol of reducing sugar equivalents per 1 mL during 1 min, at pH 5.0 and 40 °C, under applied experimental conditions. For normalization—the sugar background control (sugars contained in the sample – the substrate plus the sample without incubation at 40 °C) was run simultaneously and allowed for during calculations.

#### MicroSNT

After incubation of the substrate and the enzyme-containing sample at 40 °C, the reactions were stopped by addition of 10–40 μL of Nelson’s copper reagent I + II and heated up to 75–99.9 °C, over 1–10 min. Samples were cooled down to the room temperature (RT) and 80 μL of each sample was transferred using a multichannel pipette into a 96-well flat-bottom MTP compatible with the plate reader and mixed with 40 μL of the arsenate-molybdate reagent. Covered MTP plate was then incubated at RT in a rotary shaker at 250 rpm for 5 min. At the optimization step, the following levels of variables were considered: heating temperature 75, 80, 85, 90, 95, and 99.9 °C; heating time 1, 2, 3, 4, 5, 6, 7, 8, 9, and 10 min; volume of Nelson’s copper reagent 10, 20, 30, and 40 μl. The concentration of background sugars contained within the culture medium supernatants was determined and taken into account in the calculations.

#### MacroSNT

The principle of the macroassay was analogous as described above for the microassay. After incubation at 40 °C, 250 μL of the reaction mixture was combined with equal volume of Nelson’s copper reagent and boiled in the thermalblock for 20 min. After cooling down of the reaction mixture, 250 μL of arsenate-molybdate reagent was added.

### Starch iodine test

The micro- and macro-SIT assays were developed based on the previous methods described in (Fuwa 1954; Xiao et al. 2006). Polymer starch content was monitored by measuring of the staining value of starch-iodine chromogenic complexes versus a standard curve. The following test-specific reagents were used in the protocol: 5 mM I_2_ in 5 mM KI, and 1 M HCl.

Standard curves for SIT micro- and macro-volume protocols were conducted with liquefied starch solutions of defined concentrations (0–2 mg/mL) in 100 mM acetate buffer, pH 5.0. Absorbance spectrum for the customized microSIT assay was conducted using 2 mg/mL starch solution, within the wavelength range λ = 400–700 nm, every 10 nm.

#### One activity unit

This corresponds to the amount of an enzyme that contributes to decrease in starch-iodine staining value equivalent to 1 mg of starch per 1 mL, during 1 min at pH 5.0 and 40 °C, under applied experimental conditions. In calculations, the staining value of starch-iodine complexes that remained in the reaction mixture after digestion was subtracted from the staining value of the total starch-iodine complexes contained in the control samples (the substrate plus the enzyme sample, arrested with 1 M HCl prior to the sample addition).

#### MicroSIT

Incubation time of the substrate and the enzyme-containing sample (20 μL each) varied as described above. Reaction was stopped by adding 10 μL of 1 M HCl, and the remaining starch was stained by adding 50 μL of iodine solution. The color development was carried out in a transparent flat-bottomed 96-well microplate incubated for 5 min in a rotary shaker at 250 rpm at RT, followed by immediate measurement of the absorbance at 580 nm wavelength in the plate reader.

#### MacroSIT

The macroSIT assay relied on the same principles as described for the microSIT, with modifications regarding the reagents and samples volumes: the substrate and the sample were mixed at equal volumes (200 μL) upon initiation of the reaction. The reaction was terminated by addition of 100 μL of 1 M HCl. The remaining undigested starch was stained by addition of 500 μL of iodine. The staining value was measured after 5 min incubation at the RT.

### Statistical analysis

All the results were calculated and expressed as means ± standard deviation (±SD) of 3 or 4 replicates, as indicated above. Linear regression models were developed using Statistica 13.0 software (Statsoft, USA). Generalized linear model (GLM) was used for comparison of slopes coefficients and determination of statistical importance of the differences between the slopes. Statistical importance of the differences between compared sets of data was analyzed using one-way analysis of variance (ANOVA) and Tukey’s multiple comparison tests (Statistica; Statsoft, USA). The level of significance was set at (*p* = 0.05). Detailed results of more extensive statistical analysis are provided as Supplementary Material (Table S1). Graphical presentation of the obtained data was done using Microsoft Excel 2013 software.

## Results

### General outline of the experiments

**Fig. 1:**
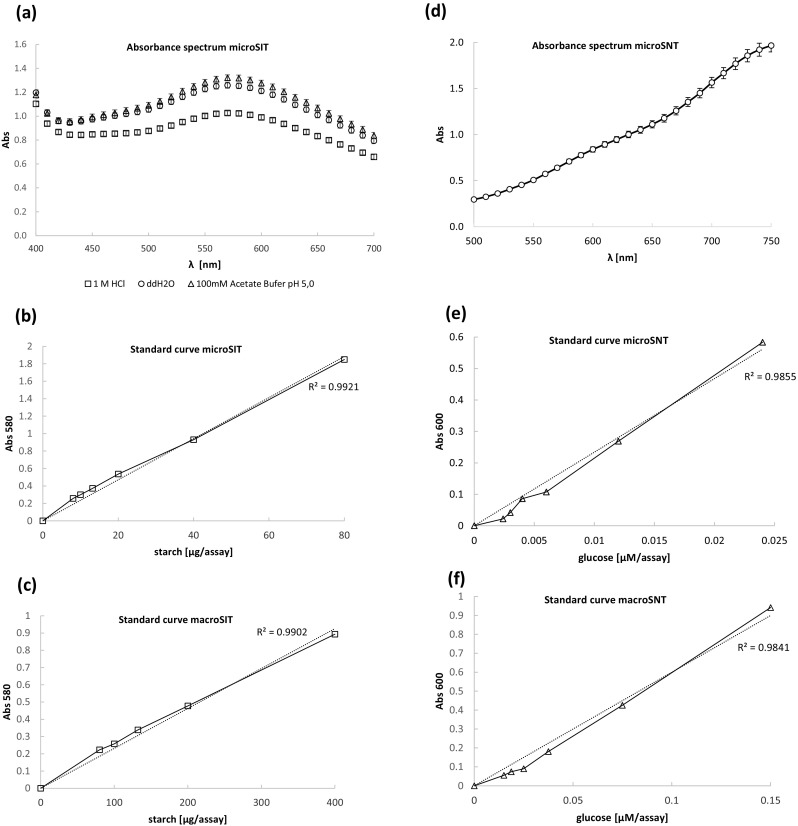
Absorbance spectra and standard curves of the assays. **a** Absorbance spectrum of chromogenic complex obtained in microSIT assay, analyzed as described in sections 2.3. and 2.3.2 after addition of 10 μL of 1 M HCl (□), or ddH2O(○), or 100 mM acetate buffer pH 5.0 (_Δ_); **d** Absorbance spectrum of chromogenic complex obtained in microSNT assay, analyzed as described in section 2.3.1. Standard curves for microSIT (**b**), macroSIT (**c**), microSNT (**e**), macroSNT (**f**) assay. X axis: λ wavelength [nm] (**a**, **d**); concentration of starch [μg/assay] (**b**, **c**); concentration of glucose [μmol/assay] (**e**, **f**); Y axis: absorbance values at: various wavelengths (**a**, **d**), at 580 nm (**b**, **c**), and at 600 nm (**e**, **f**) wavelength. The two curves in (**b**, **c**, **e**, **f**) correspond to the experimental results and the trend curve, provided with R^2^ coefficient. Error bars indicate ±SD from triplicates

The research presented in this article can be roughly divided into two stages: (i) the microassays development and optimization stage, and (ii) validation of the optimized microassays performance in comparison to their macro-volume counterparts using pure and crude enzymatic preparations. The first stage was conducted using standard solutions of the reaction substrates without treatment with enzymatic preparations (SIT, 0.2–2 mg of starch/mL of assayed sample; 0.008–0.08 mg/microassay and 0.08–0.8 mg/macroassay; SNT(moles), 0.06–0.6 mM of reducing sugar in the assayed sample; 0.0024–0.024 μM/microassay and 0.015–0.15 μM/macroassay; SNT(grams), 0.0108–0.108 mg of reducing sugar/mL of the assayed sample; 0.432–4.32 μg of reducing sugar/microassay and 2.7–27 μg of reducing sugar/macroassay) (Fig. 1b–f), with the exception of testing linearity of starch digestion at prolonged incubation times (up to 120 min; macroSNT), where decomposition of the substrate by alpha-amylase (culture medium supernatant of recombinant strain) was measured (Fig. 5). In the second stage of this research, either purified enzymatic preparations or crude culturing media containing the enzymes were used for hydrolysis of starch contained in the buffered substrate solution.

### Development of microSIT assay

Our initial experiments were focused on customization of a classical starch-iodine macro-assay (Fuwa 1954) and an MTP-based starch-iodine assay (Xiao et al. 2006) to the thermalcycler-based protocol. It turned out that the previous protocols could be adopted to the new format (twofold lower volumes of the reaction when compared to the MTP-based assay) with excellent linearity (R^2^ = 0.9921; *p* < 0.0001) in the desired range (8–80 μg starch/microassay), and the absorbance values below 2.0 (Fig. 1b; Table S1). In comparison, the macro-volume SIT assay was linear between 80 and 400 μg/macroassay (R^2^ = 0.9902, *p* < 0.0001, tested 80–800 μg, but linearity was lost above 400 μg) (Fig. 1c; Table S1). We observed that both micro- and macro-SIT assays share high positive correlation in the ratio between starch content (μg/assay) and absorbance readout (Abs 580 nm; *r* = 0.9458), but the microassay is ~ tenfold more sensitive in detection of undigested starch helices.

Subsequently, we verified whether the addition of HCl to the reaction mixture, which is required for stopping the reaction and lowering pH of the mixture, changes the maximum absorbance value of the starch-iodine complexes. To this end, we tracked absorbance spectrum of the chromogenic complexes upon addition of 1 M HCl, acetate buffer, and water. As demonstrated in (Fig. 1a), the maximum absorbance was observed at 580 nm, implying that the absorbance spectrum was not changed, and HCl constitute an important stabilizer of the chromogenic starch-iodine complex without affecting its spectral properties.

Altogether, due to good linearity in the desired range of the undigested substrate concentration and not altered absorbance maximum upon HCl addition, we concluded that the micro-volume method operates well and can be further used in the second stage of this study.

### Development of microSNT assay

**Fig. 2:**
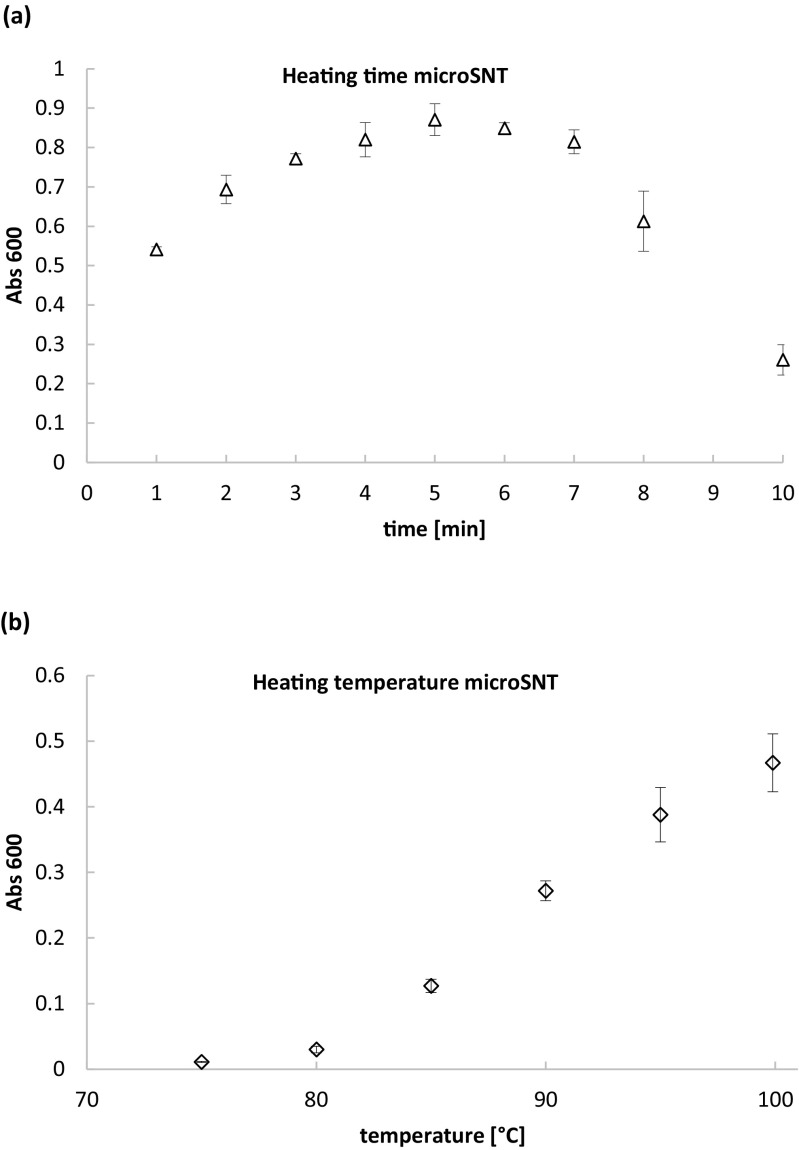
Effect of duration (**a**) and temperature (**b**) applied to a heating step in microSNT assay on copper ions reduction degree. The test was conducted as described in sections 2.3. and 2.3.1 using 0.6 mM glucose solution. X axis: duration of the heating step [min] (**a**), temperature of heating [°C] (**b**), Y axis: absorbance values at 600 nm wavelength (Abs 600), reflecting degree of the copper ions reduction. Error bars indicate ±SD from triplicates

In the next step of the present study, we focused on optimization of the Somogyi-Nelson alkaline copper protocol in the micro-volume format. According to the data presented in (Fig. 2a, b; Table S1), as little as 5 min incubation at 99.9 °C is sufficient for full reduction of the copper ions for here applied reaction volumes. In fact, boiling time within the range between 4 and 7 min was shown to give comparable results (no statistically important differences at *p* < 0.05); however, 5 min was found to be optimal time due to the highest value of absorbance at 600 nm. Heating at 99.9 °C was shown to have strong positive effect on the absorbance readouts (Abs 600 nm), significantly different from those obtained upon boiling at any other lower temperature (*p* < 0.05).

**Fig. 3:**
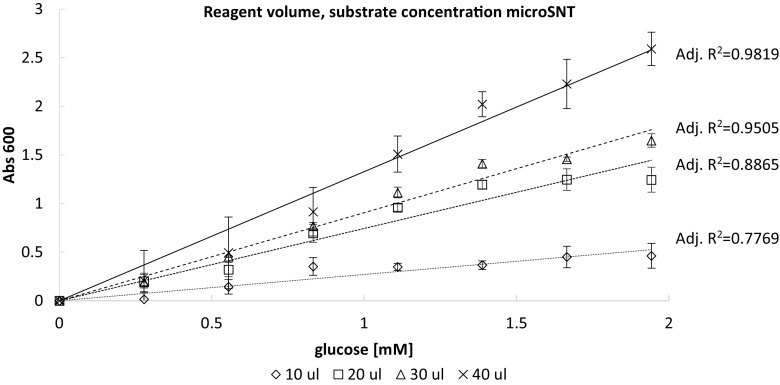
Effect of Nelson’s copper reagent volume on course, linearity and range of the standard curve in microSNT assay. The test was conducted using standard glucose solution covering the concentrations ranging from 0.28 to 1.94 mM according to the methodology described in sections 2.2. and 2.3.1. Diamonds (◊), squares (□), triangles (Δ) and crosses (x) indicate tests conducted with 10, 20, 30 and 40 μL of Nelson’s reagent, respectively. Continuous, wider dashed, narrower dashed, and dotted black lines indicate regression curves for microSNT assay with 10, 20, 30, and 40 μL of Nelson’s copper reagent, respectively. Given Adj. R^2^ coefficients (calculated as described in section 2.3.3) are provided in sequence starting from the highest (top) to the lowest (bottom) volume of Nelson’s copper reagent; for accompanying statistical analysis see Table S1. X axis: glucose concentration [mM]; Y axis: absorbance values at 600 nm wavelength (Abs 600), reflecting degree of the copper ions reduction. Error bars indicate ±SD from triplicates

For the initial optimization steps described above, the volumes of the test-specific reagents were decreased proportionally to the original protocol (Nelson 1944), maintaining constant ratios between them. However, we decided to test whether the amount of the crucial copper reagent should be corrected due to the novel assay conditions and format. Thus, we tracked the course of the standard curves (concentrations 0.28–1.94 mM of glucose) depending on the volume of Nelson’s copper reagent used (10–40 μL). During this experiment, the volume of glucose standard solutions remained constant at 40 μL. As it can be seen in (Fig. 3; Table S1), reduction in the reagent volume to 10 μL resulted in the weakest correlation between the amount of reducing sugar and the absorbance value (Adj. R^2^ = 0.7769; *p* < 0.01). When Nelson’s reagent was provided into the reaction mixture at 20 μL, the relation was linear up to 1.39 mM of glucose, whereas above this concentration—no changes in the absorbance value were observed, implying deficiency of the copper reagent (Adj. R^2^ = 0.8865; *p* < 0.001). On the other hand, the standard curve presented satisfactory linearity in the whole analyzed range of glucose concentrations, when either 30 or 40 μL of Nelson’s reagent was used. For these variants, the correlation between the absorbance readouts and the glucose concentrations was of Adj. R^2^ = 0.9505 (*p* < 0.0005) and Adj. R^2^ = 0.9819 (*p* < 0.00001), respectively (see also Table S1). Nonetheless, the best standard curve linearity and the broadest substrate concentration range were observed, when 40 μL of Nelson’s copper reagent was used (ratio 1:1 with the substrate and the sample mixture), as it was initially presumed and used in the initial optimization tests.

Based on the obtained results, we prepared a standard curve for the microSNT assay, adopting the conditions determined during the optimization tests (Fig. 1e; Table S1). As it can be seen in (Fig. 1e), the method is characterized by high linearity starting from 0.0024 to 0.024 μmol/assay of the reducing sugar equivalents (R^2^ = 0.9855; *p* < 0.0001). Standard curve demonstrates that the sensitivity of the assay was further increased when compared to the MTP-based method, since as little as 0.432 μg of glucose (10.8 μg/mL of analyzed sample) could be detected by the here developed protocol.

Additionally, we checked, whether modifications introduced to the new microSNT protocol influenced the absorbance spectrum of the chromogenic complex (Fig. 1d) and how the linear range of the microSNT protocol corresponds with its macro-volume counterpart (Fig. 1f). In terms of the linearity range for the micro- and macro-SNT methods, both methods were tested on the same set of standard solutions, ranging from 0.06 to 0.6 mM glucose, but the microassay operated well at significantly lower concentrations of glucose in the reaction mixture (microSNT 0.0024–0.024 μM per assay, macroSNT 0.015–0.15 μM per assay, Fig. 1e, f; Table S1). Finally, here, optimized microSNT method showed high positive correlation with its classical, macro-volume counterpart (*r* = 0.9979) in terms of ratio between the absorbance readouts (Abs 600 nm) and reducing sugar concentration in the analyzed samples.

### Validation and cross-testing of the assays with purified and crude enzymatic preparations

**Fig. 4:**
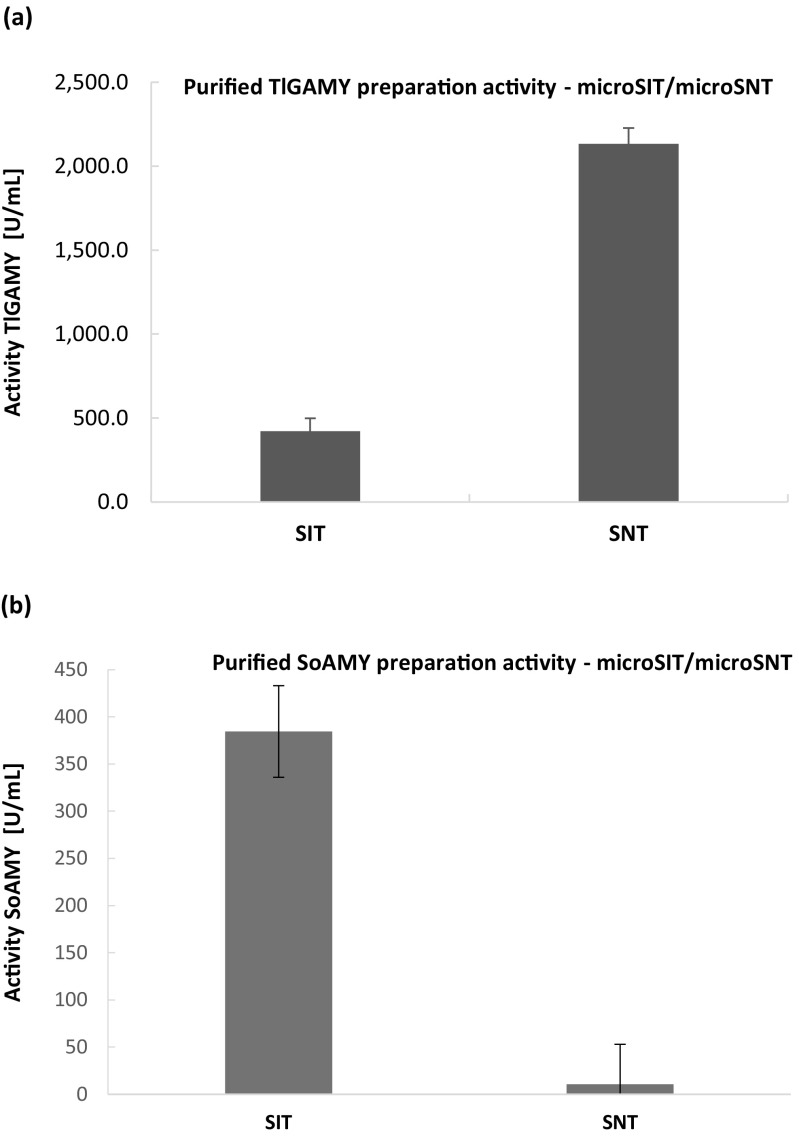
Comparison of microSIT and microSNT assays in determination of amylolytic activity of TlGAMY glucoamylase (**a**) and SoAMY alpha-amylase (**b**). The tests were conducted according to methodologies described in sections 2.3., 2.3.1., and 2.3.2 using purified enzymatic preparations containing SoAMY and TlGAMY amylases. X axis: type of the microassay; Y axis: activity values [U/mL] determined according to definitions given in sections 2.3.1. and 2.3.2 for SNT and SIT assay, respectively. Error bars indicate ±SD from triplicates

Having the described above microassays adopted to the thermalcycler-based protocol, we tested their performance with purified enzymatic preparations containing separately alpha-amylase and glucoamylase. The rationale behind this experiment was to check whether the methods show specificity towards assaying different types of amylolytic activity. As demonstrated by the data presented in (Fig. 4a, b), microSIT assay was more sensitive in detection of endoglucanase activity, while microSNT—exoglucanase.

**Fig. 5:**
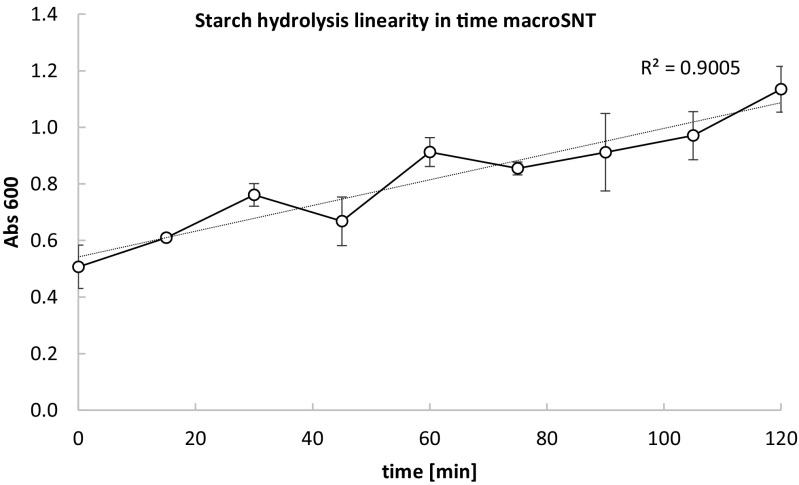
Linearity of starch decomposition by SoAMY alpha-amylase contained in crude media supernatants at prolonged incubation (up to 120 min). The test was conducted according to macroSNT methodology described in sections 2.3. and 2.3.1. using crude medium supernatants containing SoAMY alpha-amylase expressed and secreted by Po1g-derived *Y. lipolytica* strain, transformed with pYLSC-SoAMY genetic construct. The two curves correspond to the experimental results and the trend curve, provided with R^2^ coefficient, reflecting linearity of the reaction throughout the incubation time. X axis: time of incubation [min]; Y axis: absorbance values at 600 nm wavelength (Abs 600), reflecting degree of the copper ions reduction. Error bars indicate ±SD from triplicates

**Fig. 6:**
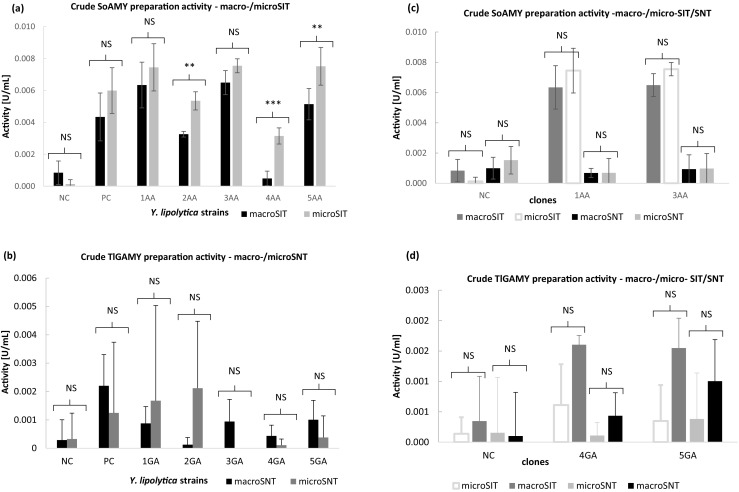
Comparison of the assays performance in determination of amylolytic activities in crude medium supernatants of *Y. lipolytica* cultures. SoAMY alpha-amylase activity (**a**, **c**) determined via macro-/microSIT (**a**) and macro−/micro-SIT/SNT (**c**) assays in crude culture supernatants of *Y. lipolytica* strains; TlGAMY glucoamylase activity (**b**, **d**) determined via macro-/microSNT (**b**) and macro-/micro-SIT/SNT (**d**) assays in crude culture supernatants of *Y. lipolytica* strains; NC negative control, Po1g strain; PC positive control, Po1g-derivative bearing pYLSC-*SoAMY* (**a**, **c**) or pYLSC-*TlGAMY* (**b**, **d**) genetic construction; strains 1–5 AA – Po1d-derivatives bearing Golden Gate Assembly cassette (GGA)-*SoAMY* construction (**a**, **c**); strains 1-5GA – Po1d-derivatives bearing GGA-*TlGAMY* constrution (**b**, **d**); Cultures were conducted as described in section 2.2., and enzymatic assays—2.3. X axis: different crude supernatant preparations containing either SoAMY alpha-amylase (**a**, **c**) or TlGAMY glucoamylase (**b**, **d**) withdrawn from different *Y. lipolytica* strains culture; Y axis: activity values [U/mL] determined according to definitions given in sections 2.3.1. and 2.3.2 for SNT and SIT assay, respectively. Error bars indicate ±SD from triplicates. Statistical importance of the observed differences was analyzed as described in section 2.3.3; * denotes *p* < 0.05, ** *p* < 0.01, *** *p* < 0.005, NS not significant

In the final stage of the current research, we investigated performance of the new SIT/SNT microassays in comparison to their macro-volume counterparts in analysis of amylolytic activity contained in raw medium samples withdrawn from the recombinant yeast cultures. *Y. lipolytica* strains, bearing separately either *SoAMY* or *TlGAMY* genes under the control of strong promoter, secreted the recombinant proteins to the cultivation medium (Celińska et al. 2018). In this experiment, crude medium supernatants were directly used as enzymatic preparations for hydrolysis of starch, without any purification procedures. Since such crude preparations were characterized by lower activity than the purified, concentrated enzymatic preparations, longer incubation time with the substrate was required. Linearity of the starch decomposition throughout the adopted incubation period was validated, and the results are presented in Fig. 5. Based on the results obtained with the purified enzymatic preparations (Fig. 4a, b), *Y. lipolytica SoAMY+* cultures were initially analyzed via SIT method (Fig. 6a), while *Y. lipolytica TlGAMY+*—via SNT micro- and macro- protocols (Fig. 6b). SIT assays in micro- and macro-scale showed very high positive correlation in the Abs 580 nm readouts (*r* = 0.91) and in most cases—no statistically important differences in the results between micro/macro-SIT. Such an outcome perfectly validates here developed thermalcycler-based microSIT assay as a tool for high-throughput screening of amylolytic activities in microbial cultures. On the other hand, we observed that SNT protocols, both macro- and micro-formats, were less suitable for analysis of raw culture media samples (Fig. 6b). First of all, we observed high deviation between the replicates and high background absorbance in the negative controls (despite of normalization of the results, as described in the Materials and Methods section) and weak positive response from the positive control sample (strain with known, high glucoamylase activity). The recombinant strains uncharacterized previously with respect to the TlGAMY activity level gave very poor results when analyzed through the SNT assays, irrespective of the reaction volume. The most probable factors contributing to the poor performance of SNT assays with raw culture media are disturbing agents introduced with the raw culture supernatants (components, pH).

In order to finally address the question of suitability of the novel microassays as tools for rapid amylolytic activities screening in crude culture media, we did cross-assaying of samples via all macro-/micro- SIT/SNT tests (Fig. 6c, d). In terms of determination of SoAMY activity in the crude culture media, SIT assays gave highly correlating readouts between macro- and micro-format, nicely standing out of the negative control (especially microSIT), with acceptable level of deviation, irrespective of the scale. On the other hand, SNT assays failed to detect alpha-amylolytic activity in the crude culture media, which could be explained with its lower sensitivity towards this type of amylolytic activity, as shown in (Fig. 4a). Nevertheless, upon assaying TlGAMY glucoamylase activity in the crude culture media, again, SNT assays gave poor results, with high background activity level in the negative controls. Surprisingly, micro-/macro-SIT assays were in this case more sensitive to glucoamylase activity which, taken together with the results presented in (Fig. 4b), clearly show that constituents of the crude culture media disallow accurate assessment of TlGAMY activity. This in turn, also explains poor performance of macro-/micro-SNT assays shown in (Fig. 6b).

## Discussion

This study reports customization of standard macro-volume and MTP-based assays for amylolytic activity determination to the micro-volume format protocols employing a thermalcycler, and validation thereof. Both assays under study SIT and SNT are commonly known standards of assessing amylolytic activities or reducing sugar content in the samples. The protocols were initially developed as “macro-volume” assays (Nelson 1944; Fuwa 1954), and later-on adopted as MTP-based assays (Xiao et al. 2006; Shao and Lin 2018). In order to improve screening capacity of the methods, in the present study, we further miniaturized the assays to 96-well PCR plates format and adopted thermalcycler for incubation and heating of the samples.

In terms of linearity range and sensitivity, here, developed thermalcycler-based microSIT assay well corresponds with the previously developed MTP-based protocol by Xiao et al. (2006). In the MTP-based micro-assay (Xiao et al. 2006), the linear range (R^2^ = 0.9998) of the MTP-based assay was corresponding; however, the absorbance values above 2.0 were accepted. On the other hand, in the current study, we further reduced the reaction volume as well as the time required for the assay by twofold, when compared to the MTP-based assay by (Xiao et al. 2006). When it comes to the macro-volume SIT assays, in the original protocol by (Fuwa 1954), the linearity was investigated up to 5 mg of amylose in the assayed sample and showed excellent linearity; however, the volume of the reaction mixture was 250-fold higher, than of the here presented macro-assay (conducted in 1 mL), which could impose a serious challenge upon analysis of multiple samples.

In the original macro-volume SIT protocol (Fuwa 1954), acetic acid was added in order to terminate hydrolysis reaction. In another study (Pimstone 1964), the reaction was terminated by addition of sulfuric acid. In the microassay by Xiao et al. (2006)), the reaction was stopped by addition of 1 M HCl, as it was in the current study for both macro- and micro-volume SIT assay. Apart from termination of the enzymatic reaction, addition of acid is necessary to decrease pH. It is known that under lower pH values, starch-iodine chromogenic complexes are more stable and enhanced in color (Pimstone 1964). Starch-iodine complexes are intrinsically prone to photo-bleaching/higher-pH-bleaching phenomenon. Since we also encountered the problem of the complex instability, especially when the crude culture media samples were used for starch hydrolysis (higher pH), we added HCl to the reaction mixtures. HCl addition changes the acidity of the reaction mixture which affects iodide ions binding, and ultimately may influence the absorbance of the chromogenic starch-iodine complex. To address the question whether addition of HCl affects absorbance spectrum of the chromogenic starch-iodine complexes, we tracked its absorbance spectrum upon addition of HCl, acetate buffer, and water. Our results demonstrated that the absorbance spectrum of the complexes remained unchanged by addition of HCl, while we noted that stability of the color was improved, which altogether indicates positive effect of acidification of the microSIT reactions.

Customization of Somogyi-Nelson alkaline copper protocol to micro-volume thermalcycler-based assay required more intensive investigation of the optimal reaction conditions. As previously stated, macroSNT assay is manageable when a small number of samples is processed, but it becomes problematic when the number of sample increases (Green et al. 1989). It was also raised that a boiling step included in the original protocol, being crucial for reduction of the copper ions, was the limiting factor in adaptation of the macroSNT to the MTP format. This statement was the key rationale behind our attempts of adopting the MTP-based assay to the thermalcycler-based protocol, which enables high-temperature heating of the samples. In the MTP-based protocol by Green et al. (1989), the boiling step was replaced by prolonged incubation at 80 °C for 30 min, since MTPs could not be used at higher temperatures. The original SNT macroassay requires 10–20 min of heating, depending on the sample composition (Nelson 1944; Somogyi 1952). It was also confirmed in a study by Marais et al. (1966) that 20 min boiling was required to reach complete reduction of the copper reagent by glucose in a standard macroassay. In the here developed method, due to adaptation of a thermalcycler and compatible, heat-resistant plates, the high-temperature step could be easily included in the protocol. Considering the solution proposed by Green et al. (1989) (lower heating temperature), the original protocol by Nelson (Nelson 1944), and possibilities of the employed equipment (max. 99.9 °C in a standard thermalcycler), we tested different heating temperatures, and analyzed which combination of time and temperature allows for sufficient reduction of the copper ions—giving the highest absorbance readouts at 600 nm wavelength. In the thermalcycler-based protocol developed in this study, this time could be reduced fourfold, when compared to the macroassay. When compared to the MTP-based protocol presented in (Green et al. 1989), the reduction in time required for this step is even more pronounced (sixfold), as in that report; the samples were incubated over 30 min at 80 °C. The recently developed SNT microassay protocol, conducted in MTPs, again required 20 min of boiling for complete copper reduction (Shao and Lin 2018).

Additionally, in the present study, we checked whether modifications introduced to the new microSNT protocol influenced the absorbance spectrum of the chromogenic complex (Fig. 1d) and how the linear range of the microSNT protocol corresponds with its macro-volume counterpart (Fig. 1f). The absorbance spectrum observed for the chromogenic complex obtained via microSNT protocol was identical as the spectrum observed for the complex obtained via MTP-based assay conducted with defined sugars solutions (Shao and Lin 2018), implying that it was not affected by the incorporated modifications. Additionally, Shao and Lin (2018) recommended to conduct absorbance readouts at 600 nm, at which the discrepancy in the maximum absorbance of different types of sugars (obviously contained in the enzyme-treated sample in this study) was less than 0.05, but increased to 0.25 while measuring at 750 nm. In that report, it was also recommended to conduct the measurements of the color complex at 600 nm, and not at higher wavelengths, which ensures good detection range and the acceptable sensitivity. Moreover, the time required to develop a color for measuring at 600 nm was more than fivefold shorter, than for measuring at 750 nm (Marais et al. 1966). Considering linearity and sensitivity of the thermalcycler-based microSNT test, it was further improved, when compared to the previous MTP-based protocols. In (Green et al. 1989), the minimal amount of glucose detectable by the MTP-based assay was 100 μg/mL, so the sensitivity of here presented microSNT assay was enhanced tenfold. Moreover, the amount of sample was also further reduced from 25 μL in (Green et al. 1989) (total reaction volume 200 μL) to 20 μL (total reaction volume 120 μL). Importantly, we did not observed formation of precipitates in our microSNT (as well as macroSNT) assays when either glucose or purified enzymatic preparations were used, although it was claimed an inherent trait of the SNT method (Green et al. 1989).

After optimization of technical parameters of the two micro-assays, we moved to their validation with enzymatic preparations. Primarily, purified enzymatic preparations containing endoglucanase, represented by SoAMY alpha-amylase, and exoglucanase (TlGAMY glucoamylase) were analyzed by both tests. Expectedly, microSIT assay performed better with endoglucanase, while microSNT gave higher readouts with exoglucanase. Such an outcome was expected, considering the two enzymes’ mode of action—endo- and exo-glucanase, and characteristics of the two assays. Glucoamylase is more efficient in generation of reducing sugar ends, which are detected in SNT test, while alpha-amylase is more efficient in disruption of starch helices, which form chromogenic complexes with iodine in the SIT method. This observation was earlier thoroughly discussed in (Xiao et al. 2006) upon comparison of SIT and DNS methods towards alpha-amylase activity assessment, where the results of microSIT assay were five times higher than those obtained with microDNS method (determining reducing sugars concentration). Noteworthy, as thoroughly discussed in (Shao and Lin 2018), the DNS assay is now considered to be an inaccurate method for measuring reducing sugars of mixed carbohydrates (for details, please refer to that paper).

Significant fraction of reported studies on development of enzymatic activity assays come down to validation of the protocol with standard solutions of the substrates or purified enzymatic preparations. From our experience, raw samples withdrawn from complex matrices (food, biomass samples, microbial cultures etc.) may impose significant difficulties in assaying, due to their characteristics. Therefore, in order to finally address the question of suitability of the novel microassays for rapid screening of amylolytic activities or starch/reducing sugar content in complex matrices, we did cross-assaying of crude culture media samples via all macro-/micro-SIT/SNT tests. The obtained results demonstrated that SIT assay, irrespective of the format, was resistant towards impurities contained in the crude samples. On the other hand, microSNT assay performed poorly with crude samples, as illustrated by high background level in the negative controls and high deviation between the readouts. One of the factors contributing to such an outcome could be formation of the insoluble precipitate encountered in the reactions with the crude culture media supernatants. In the macro-scale, the precipitate could be removed by simple centrifugation of the processed samples, as we did previously (Celińska et al. 2015, 2016a, b, 2017). Nevertheless, additional step obviously disrupts rapid character of the methods, and thus disqualifies it from high-throughput screening of crude culture media. As proposed earlier, formation of this insoluble precipitate could be alleviated by measuring the color development by reflectance rather than absorbance (Green et al. 1989); however, it requires specific equipment, which is not available in our facility. Still, having such possibility makes the microSNT assay worth testing again with crude enzymatic extracts, as it was proved to operate perfectly with clear enzymatic suspensions containing glucoamylase activity. Altogether we conclude that while microSIT protocol performed equally well at the stage of the method optimization with standard solutions, as well as at the stage of testing the method with starch hydrolysates generated by enzymatic preparations, the microSNT did not impose any complications, until crude culture medium was used as the hydrolyzing agent. Previous studies on development of MTP-based microSNT protocols (Green et al. 1989; Shao and Lin 2018) presented optimized assays, validated with different types of reducing sugar standard solutions. Our current study demonstrates methods that were tested with crude samples, containing heterogeneous mixtures of sugars, which are present in various matrices, like foods or enzymatically processed hydrolysates.

In summary, the present study describes development of two microassays having complementary scope of specificities—SIT—more appropriate for endoglucanases, and SNT—more specific for exoglucanases. We suggest that here, developed microSNT method can be successfully used with relatively clear enzymatic hydrolysates composed of heterogeneous mixture of mono- and oligosaccharides. On the other hand, microSIT is suitable for testing both purified or crude enzymatic preparations with respect to their amylolytic activity. Moreover, the microSIT test, while more sensitive for determination of endoglucanase activity, is still operable with exoglucanase-treated starch samples. Due to rapid, micro-volume, and high-throughput character, the two methods can assist development and engineering of novel, robust biocatalysts, as well as testing multiple samples containing heterogeneous mixtures of sugars.

## Electronic supplementary material

ESM 1 (PDF 615 kb)
