# Supplementary material for: Rapid micro-assays for amylolytic activities determination: customization and validation of the tests
Source: Appl Microbiol Biotechnol. 2019 Jan 23;103(5):2367–79. doi: 10.1007/s00253-018-09610-0 (PMC6420461; doi:10.1007/s00253-018-09610-0)

Applied Microbiology and Biotechnology

Rapid micro-assays for amylolytic activities determination: customization and validation of the tests

Monika Borkowska, Wojciech Białas, Monika Kubiak, Ewelina Celińska*

Department of Biotechnology and Food Microbiology, Poznan University of Life Sciences, ul. Wojska Polskiego 48, 60-627 Poznań, Poland

*corresponding author: E. Celińska: ewelina.celinska@up.poznan.pl, +48 61 846 6007

Supplementary Material

Table S1. Selected details of statistical analysis of the obtained results

1. **Results presented in Fig.1.b.c.e.f**

Standard curves for microSIT, macroSIT, microSNT, macroSNT – statistical analysis of matching the experimental data with the regression model

**ANOVA microSIT**					
	df	SS	MS	F	p value
Model	1	6,861872	6,861872	138,0183	1,39E-09
Error	6	0,845191	0,049717		
Total	7	7,707063			

**ANOVA macroSIT**					
	df	SS	MS	F	p value
Model	1	0,558831	0,558831	70,79859	1,82E-07
Error	6	0,134185	0,007893		
Total	7	0,693016			

**ANOVA microSNT**					
	df	SS	MS	F	p value
Model	1	0,686835299	0,686835299	1889,202778	7,23521E-19
Error	6	0,006180491	0,000363558		
Total	7	0,693015789			

**ANOVA macroSNT**					
	df	SS	MS	F	p value
Model	1	0,689251608	0,689251608	3112,835558	1,06727E-20
Error	6	0,003764181	0,000221422		
Total	7	0,693015789			

2. **Results presented in Fig. 2 (a) heating time optimization for microSNT**

HSD (honestly significant difference) Tukey test; p value = 0.05

1 – grouping

Nr podkl.	Time [min]	Absorbance [600nm] Średnie	1	2	3	4	5
9	10	0.261350				****	
1	1	0.541050					****
8	8	0.621750			****		
2	2	0.693350			****		
3	3	0.771900		****			
7	7	0.814325	****	****			
4	4	0.819975	****	****			
6	6	0.849050	****				
5	5	0.870850	****				

2 – statistical important differences (results not statistically different are given in black)

Nr podkl.	Time [min]	{1} .54105	{2} .69335	{3} .77190	{4} .81998	{5} .87085	{6} .84905	{7} .81433	{8} .62175	{9} .26135
**1**	1		0.000153	0.000151	0.000151	0.000151	0.000151	0.000151	0.018865	0.000151
2	2	0.000153		0.023955	0.000211	0.000152	0.000152	0.000284	0.050674	0.000151
3	3	0.000151	0.023955		0.401147	0.002322	0.027947	0.562152	0.000154	0.000151
4	4	0.000151	0.000211	0.401147		0.330188	0.901452	0.999999	0.000151	0.000151
5	5	0.000151	0.000152	0.002322	0.330188		0.980096	0.212162	0.000151	0.000151
6	6	0.000151	0.000152	0.027947	0.901452	0.980096		0.780979	0.000151	0.000151
7	7	0.000151	0.000284	0.562152	0.999999	0.212162	0.780979		0.000151	0.000151
8	8	0.018865	0.050674	0.000154	0.000151	0.000151	0.000151	0.000151		0.000151
9	10	0.000151	0.000151	0.000151	0.000151	0.000151	0.000151	0.000151	0.000151	

3. Results presented in Fig. 2 (b) heating temperature optimization for microSNT

HSD (honestly significant difference) Tukey test; p value = 0.05

1 – grouping

Nr podkl.	Temperature [C]	Absorbance [600nm] Średnie	1	2	3	4	5
**1**	75	0.010500	****				
2	80	0.030467	****				
3	85	0.127133		****			
4	90	0.271833			****		
5	95	0.388233				****	
6	100	0.466933					****

2 - statistical important differences (results not statistically different are given in black)

Nr podkl.	Temperature [C]	{1} .01050	{2} .03047	{3} .12713	{4} .27183	{5} .38823	{6} .46693
**1**	75		0.927261	0.001555	0.000159	0.000159	0.000159
2	80	0.927261		0.006663	0.000159	0.000159	0.000159
3	85	0.001555	0.006663		0.000335	0.000159	0.000159
4	90	0.000159	0.000159	0.000335		0.001580	0.000162
5	95	0.000159	0.000159	0.000159	0.001580		0.027434
6	100	0.000159	0.000159	0.000159	0.000162	0.027434	

4. Results presented in Fig.3. – reagents volume

**ANOVA**		DF	Sum of Squares	Mean Square	F Value	p value	Adj R2
10ul	Model	1	0,12619	0,12619	21,89423	0,00544	0,7769
	Error	5	0,02882	0,00576			
	Total	6	0,15501				
20ul	Model	1	1,05883	1,05883	47,85969	9,68E-04	0,88649
	Error	5	0,11062	0,02212			
	Total	6	1,16945				
30ul	Model	1	1,68671	1,68671	116,1122	1,19E-04	0,95046
	Error	5	0,07263	0,01453			
	Total	6	1,75935				
40ul	Model	1	4,91244	4,91244	326,8317	9,51E-06	0,98192
	Error	5	0,07515	0,01503			
	Total	6	4,98759				

Figure S1. Diagrams representing protocols under study: macroSIT, macroSNT, microSIT, microSNT. Detailed description of the protocols is provided in the main text of the article

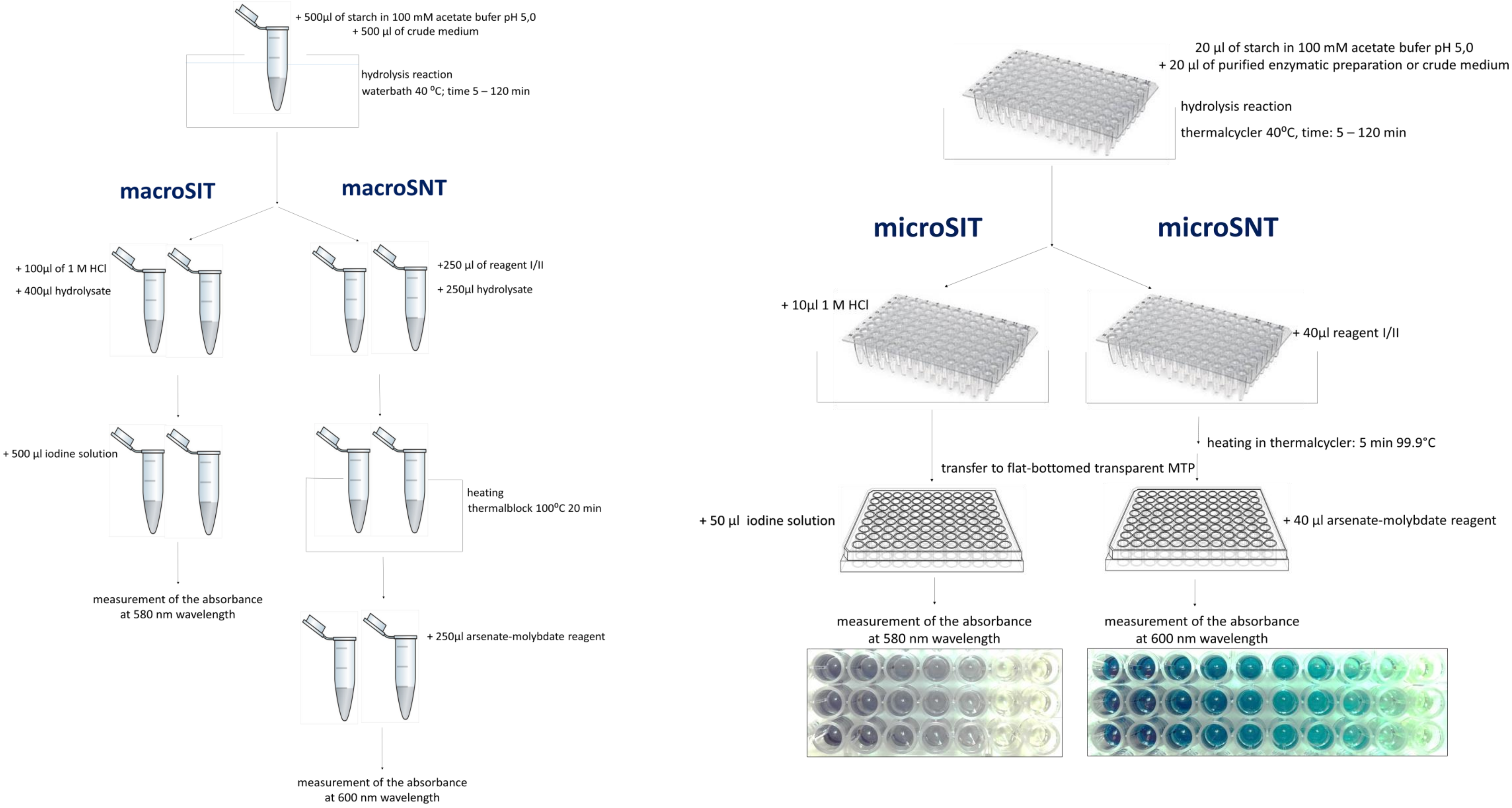

Supplement: Supplementary file 1 — (PDF 615 kb) [file 253_2018_9610_MOESM1_ESM.pdf]
